# MicroRNA-146a negatively regulates inflammation via the IRAK1/TRAF6/NF-κB signaling pathway in dry eye

**DOI:** 10.1038/s41598-023-38367-4

**Published:** 2023-07-11

**Authors:** Ruifang Han, Juan Gao, Liming Wang, Peng Hao, Xi Chen, Yuchuan Wang, Zhixin Jiang, Li Jiang, Ting Wang, Lin Zhu, Xuan Li

**Affiliations:** 1 Tianjin Key Lab of Ophthalmology and Visual Science, Tianjin Eye Hospital, Tianjin Eye Institute, Nankai University Affiliated Eye Hospital, Clinical College of Ophthalmology of Tianjin Medical University, No.4 Gansu Road, Heping District, Tianjin, 300020 China; 2 Clinical College of Ophthalmology, Tianjin Medical University, Tianjin, China

**Keywords:** Cell biology, Diseases, Molecular medicine

## Abstract

Inflammation is a key factor in the pathogenesis of dry eye disease (DED). We aimed to investigate the role of microRNA-146a (miR-146a) in regulating corneal inflammation in a mouse model of benzalkonium chloride (BAC)-induced dry eye and the TNF-α-induced NF-κB signaling pathway in human corneal epithelial cells (HCECs). A mouse model of dry eye was established by administering with BAC to BALB/c mice, and the expression of TNF-α, IL-1β, IL-6, IL-8, cyclooxygenase 2 (COX2), interleukin-1 receptor-associated kinase 1 (IRAK1) and TNF receptor-associated factor 6 (TRAF6) in the corneas of dry eye model mice was significantly increased; this was accompanied by the upregulation of miR-146a and activation of the NF-κB pathway. In vitro, TNF-α induced miR-146a expression in HCECs, while the NF-κB inhibitor SC-514 reduced the expression of miR-146a. Overexpression of miR-146a decreased the expression of IRAK1 and TRAF6, which have been identified as targets of miR-146a. Furthermore, overexpression of miR-146a suppressed NF-κB p65 translocation from the cytoplasm to the nucleus. Moreover, overexpression of miR-146a attenuated the TNF-α-induced expression of IL-6, IL-8, COX2 and intercellular adhesion molecule 1 (ICAM1), while inhibition of miR-146a exerted the opposite effect. Our results suggest that miR-146a mediates the inflammatory response in DED. MiR-146a negatively regulates inflammation in HCECs through the IRAK1/TRAF6/NF-κB pathway, and this may serve as a potential therapeutic approach for the treatment of DED.

## Introduction

Dry eye disease (DED) is a disease that is caused by instability of tear film or ocular surface damage, and it leads to eye discomfort and visual dysfunction. The clinical symptoms of DED include foreign body sensation, photophobia, itching, and visual impairment, and these symptoms reduce the quality of life of patients^1,2^. DED is a growing public health concern with a high prevalence worldwide and an increasing rate of occurrence^3^. However, the fundamental processes involved in the pathological mechanisms underlying DED remain poorly understood. Research shows that inflammation is considered to be the most important factor in the pathogenesis of DED^4,5^.

MicroRNAs (miRNAs) play roles in the posttranscriptional regulation of inflammation^6–8^. MiRNAs are a class of endogenous noncoding single-stranded small RNAs-that are 20–24 nucleotides in length. By binding to the 3′ untranslated regions (3′ UTRs) of target mRNAs, miRNAs can cause the degradation of their target mRNAs or inhibit the translation of mRNAs into proteins, thus regulating gene expression after transcription^9,10^. MicroRNA-146a (miR-146a) has been demonstrated to play an important role in the regulation of innate immunity and inflammatory responses^11–13^. MiR-146a targets TLR3 and TRAF6 to block the NF-κB pathway and negatively regulates the Coxsackievirus B (CVB)-induced inflammatory response^14^. MiR-146a mimic inhibits NF-κB-driven inflammation and leukemia progression in myeloid cells^15^. The roles of miRNAs in different cell types and pathological conditions is an area of active research. In the current study, we explored the role of miR-146a in regulating corneal inflammation.

The classic NF-κB pathway is related to inflammation and innate immunity^16^. Activation of the NF-κB pathway induces the cytokines that are related to the inflammatory response, including proinflammatory factors, chemokines, adhesion factors and enzymes that induce secondary inflammatory mediators, such as cyclooxygenase 2 (COX2). Some studies have shown that miR-146a targets TNF receptor-associated factor 6 (TRAF6) and interleukin-1 receptor-associated kinase 1(IRAK1) to regulate the NF-κB signaling pathway^17–19^. IRAK1 is a key mediator of the IL-1 receptor (IL-1R) and Toll-like receptor (TLR) pathways, which mediate the NF-κB signaling pathway^20–22^. TRAF6 is the main signal transduction molecule of the TRAF receptor superfamily and IL-1/TLR superfamily, and it mediates inflammatory and apoptotic signaling pathways^21,23^.

In the present study, we established a DED mouse model via the topical administration of benzalkonium chloride (BAC)^24,25^, and we observed the upregulation of miR-146a, IRAK1 and TRAF6, as well as the activation of the NF-κB signaling pathway in the DED mouse model. In vitro, we administered TNF-α to induce inflammatory responses in human corneal epithelial cells (HCECs). We investigated the role of miR-146a in HCEC inflammation by performing gain-of-function and loss-of-function experiments. Here, we show that miR-146 negatively regulates the inflammatory IRAK1/TRAF6/NF-κB signaling pathway in dry eye.

## Results

### The expression of miR-146a, TNF-α, IL-1β, IL-6 and IL-8 is upregulated in the corneas of dry eye model mice

**Figure 1:**
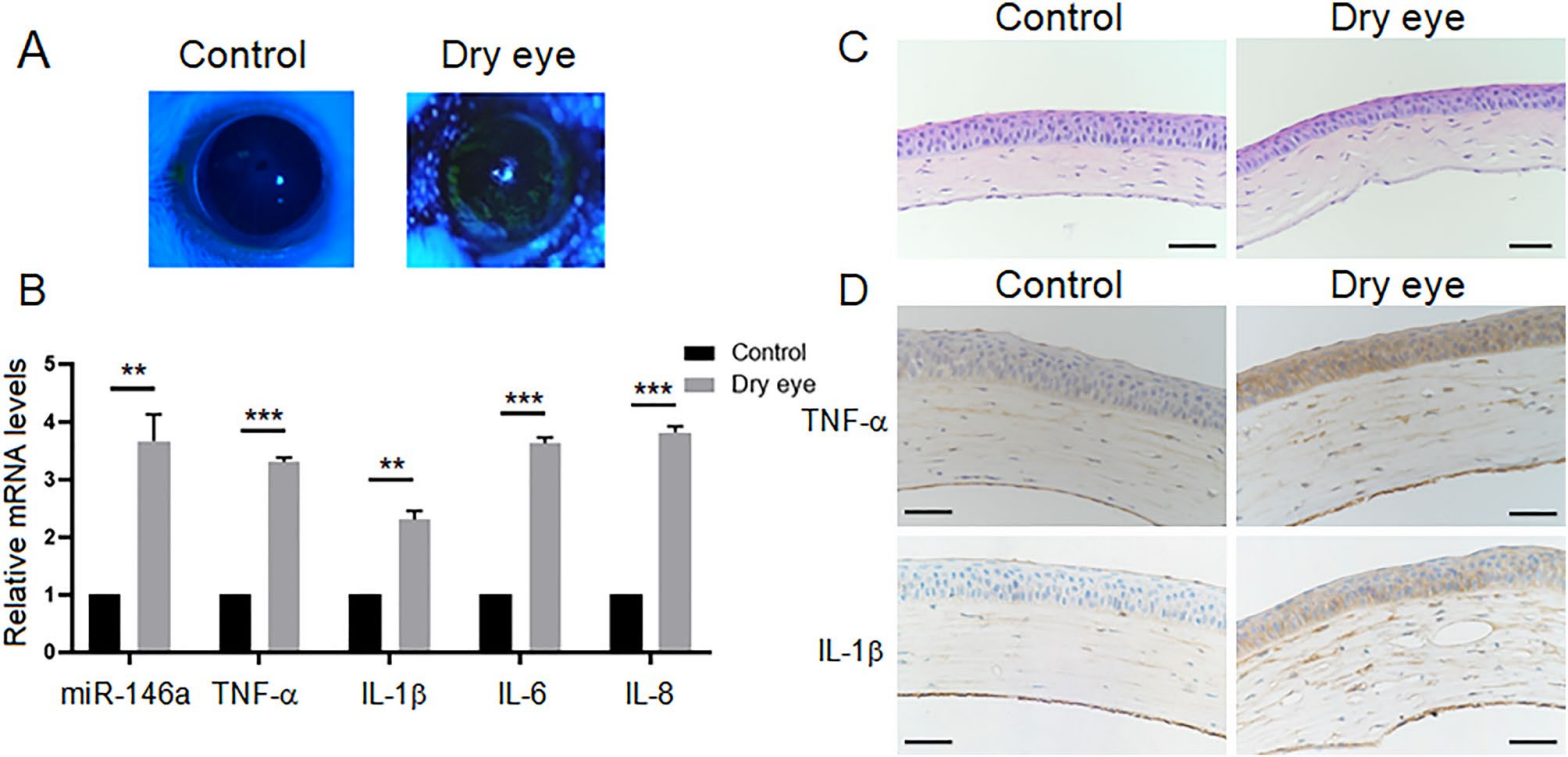
The expression of miR-146a, TNF-α, IL-1β, IL-6 and IL-8 is upregulated in the corneas of dry eye model mice. (**A**) The fluorescein staining (FLS) of the corneas in the control group (C-group) and the dry eye group (DE-group). (**B**) The mRNA expression of miR-146a, TNF-α, IL-1β, IL-6 and IL-8 in the corneas of the C-group and the DE-group was measured by real-time quantitative PCR (RT-qPCR). U6 or GAPDH was used as an endogenous control. Results are expressed as mean ± SD. **P < 0.01, ***P < 0.001. (**C**) Hematoxylin and eosin (H&E) staining showed the histological appearance of the corneas in the mice in the C-group and the mice in the DE-group. *Scale bars*: 50 μm. (D) The expression of TNF-α and IL-1β in the corneas of the C-group and the DE-group was detected by immunohistochemical staining. *Scale bars*: 50 μm.

We established a dry eye mouse model via the administration of BAC^24,25^. Three days after establishment of the dry eye mouse model, the tear film break-up time (BUT) of the dry eye group (DE-group) was 3.50 ± 0.43 s, which was markedly shorter than that of the control group (C-group) (10.45 ± 0.52 s) (*P* < 0.05). The fluorescein staining (FLS) scores of the DE-group was 13.35 ± 0.65, which was significantly higher than that of the C-group (1.25 ± 0.50) (*P* < 0.05). A large area of FLS was observed in the corneas of the DE-group, and the area of FLS in the DE-group was significantly larger than that in the C-group (Fig. 1A).

The mRNA expression of miR-146a, TNF-α, IL-1β, IL-6 and IL-8 in the corneas of dry eye model mice was measured by real-time quantitative PCR (RT-qPCR). As shown in Fig. 1B, the mRNA expression levels of miR-146a, TNF-α, IL-1β, IL-6 and IL-8 in the DE-group were upregulated by 3.67, 3.30, 2.31, 3.63 and 3.82 times, respectively, compared with those in the C-group. The results showed that the expression of miR-146a was upregulated in the corneas of dry eye model mice. Furthermore, hematoxylin and eosin (H&E) staining showed the histological appearance of the corneas in the mice in the C-group and the mice in the DE-group. In the C-group, the corneal epithelium and collagen fibers of the stroma were arranged in an orderly manner. In the DE-group, the number of corneal epithelial layers was decreased, the collagen fibers of the corneal stroma were disordered and swollen, and fibroblasts were activated (Fig. 1C). Immunohistochemical staining was used to investigate the protein expression of TNF-α and IL-1β in the corneas of the mice in the C-group and DE-group. The expression levels of TNF-α and IL-1β in the DE-group were obviously higher than those in the C-group, indicating that TNF-α and IL-1β expression was induced during corneal inflammation in dry eye model mice (Fig. 1D).

### The expression of IRAK1, TRAF6 and COX2 is upregulated and the NF-κB pathway is activated in the corneas of dry eye model mice

**Figure 2:**
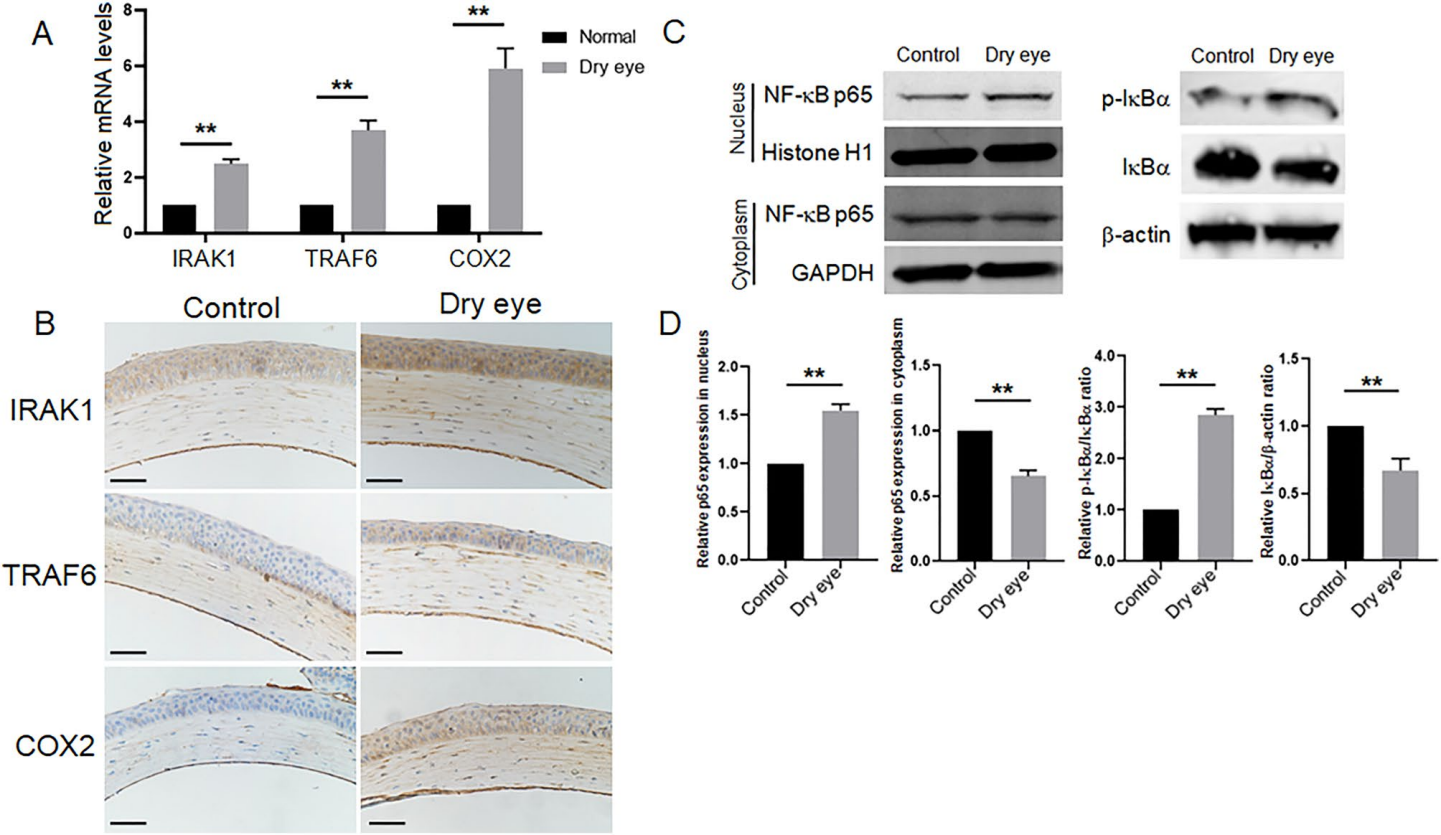
The expression of IRAK1, TRAF6 and COX2 is upregulated and the NF-κB pathway is activated in the corneas of dry eye model mice. (**A**) The mRNA expression levels of IRAK1, TRAF6 and COX2 were upregulated in the corneas of dry eye model mice measured by RT‒qPCR. GAPDH was used as an endogenous control. Results are expressed as mean ± SD. **P < 0.01. (**B**) The protein expression of IRAK1, TRAF6 and COX2 in the corneas of the C-group and the DE-group was measured by immunohistochemical staining. *Scale bars*: 50 μm. (**C**) The protein expression levels of NF-κB p65 and IκBα in the C-group and the DE-group were measured by western blotting, and the quantified data are shown in (**D**). **P < 0.01.

Studies have indicated that miR-146a plays an important role in the regulation of inflammation^12,26,27^. IRAK1 and TRAF6 are two predicted target genes of miR-146a, and this finding has been validated^17–19^. COX2 is a secondary inflammatory mediator. The mRNA expression levels of IRAK1, TRAF6 and COX2 in the corneas of dry eye model mice were measured by RT-qPCR. Compared with the C-group, the mRNA levels of IRAK1, TRAF6 and COX2 were upregulated in the DE-group (Fig. 2A).

Moreover, the protein expression of IRAK1, TRAF6 and COX2 in the corneas of mice was measured by immunohistochemical staining. The results showed that the protein expression levels of IRAK1 and TRAF6 were increased in the corneal epithelium and corneal stroma of the DE-group compared with those of the C-group, suggesting that IRAK1 and TRAF6 were involved in corneal inflammation in dry eye model mice (Fig. 2B). COX2 is a kind of inducible enzyme that is not expressed or is expressed at low levels. Its expression is induced by inflammatory mediators, and it exerts proinflammatory effects. Immunohistochemical staining results showed that the expression of COX2 in the Cgroup was low, while the expression of COX2 was obviously increased in the DE-group (Fig. 2B).

IRAK1 and TRAF6 are two well-known activators of the NF-κB pathway. We next analyzed NF-κB p65 and IκBα, which are two important proteins involved in the NF-κB pathway. The protein expression of phosphorylated IκBα upregulated in the DE-group compared with the C-group, and the expression of IκBα was downregulated in the DE-group compared with the C-group. The levels of the NF-κB p65 protein in the nucleus was increased in the DE-group compared with the C-group (Fig. 2C,D). Taken together, these data indicated that the NF-κB pathway was activated during corneal inflammation in dry eye model mice.

### The expression of miR-146a, IRAK1, TRAF6, COX2 and ICAM1 is upregulated during HCEC inflammation in vitro

**Figure 3:**
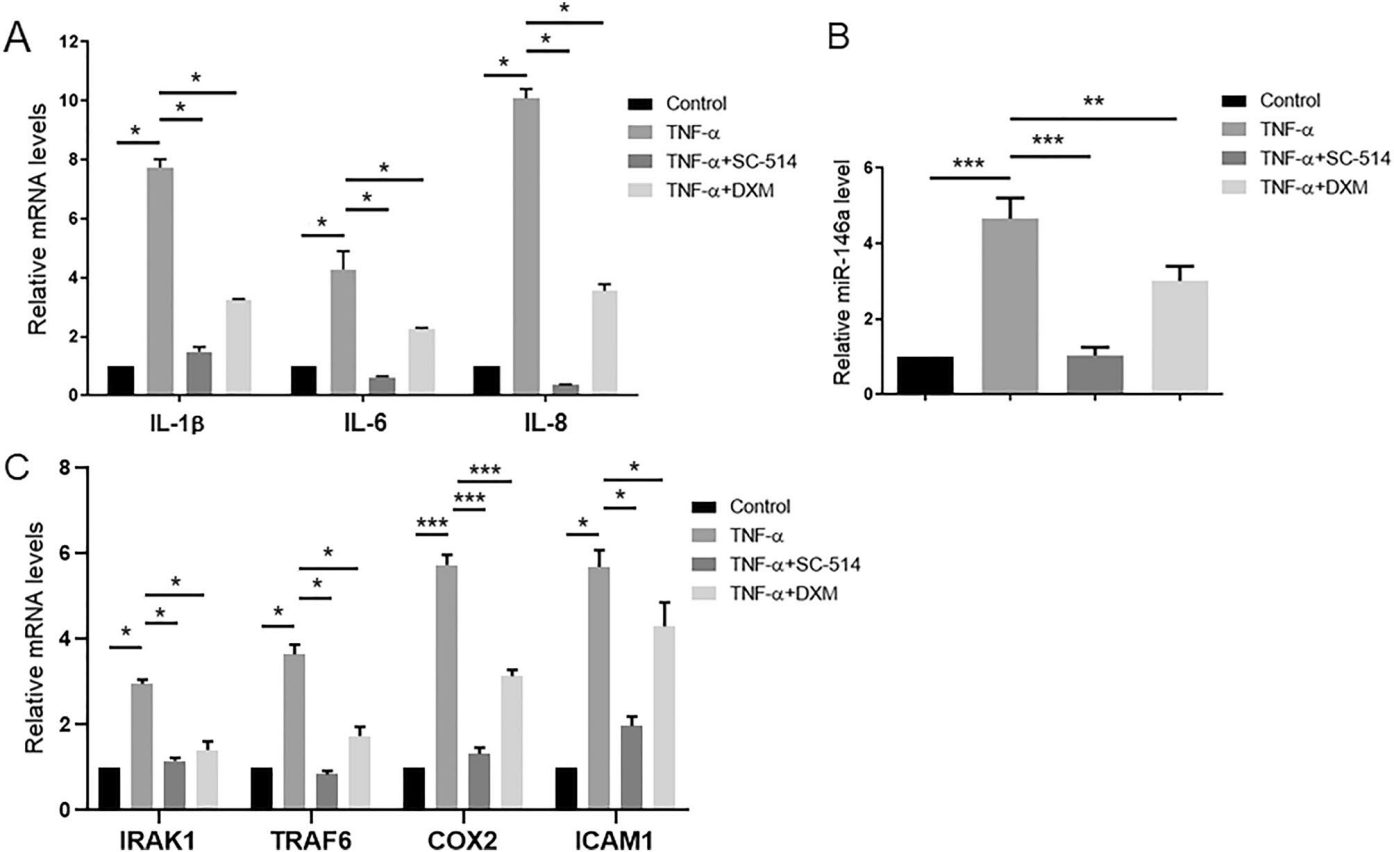
SC-514 and dexamethasone (DXM) decreased the expression of miR-146a, IRAK1, TRAF6, COX2, ICAM1 and inflammatory factors in HCEC inflammation. (**A**) The RT‒qPCR results showed that the expression of IL-1β, IL-6 and IL-8 was upregulated by TNF-α stimulation, and this effect was reversed by coincubation with SC-514 or DXM. GAPDH was used as an endogenous control. (**B**) The mRNA expression of miR-146a was upregulated by TNF-α stimulation measured by RT‒qPCR, and this effect was reversed by coincubation with SC-514 or DXM. U6 was used as an endogenous control. (**C**) The RT‒qPCR results showed that the mRNA expression of IRAK1, TRAF6, COX2 and ICAM1 was upregulated by TNF-α stimulation, and this effect was reversed by coincubation with SC-514 or DXM. GAPDH was used as an endogenous control. The results are expressed as the mean ± SD. *P < 0.05, **P < 0.01, ***P < 0.001.

To analyze the potential effects of miR-146a on HCEC inflammation, we applied TNF-α to induce inflammatory responses in HCECs. HCECs were cultured in the presence or absence of TNF-α (10 ng/ml) for 24 h after serum starvation. Dexamethasone (DXM), a glucocorticoid with a strong anti-inflammatory effect, and SC-514, an inhibitor of the NF-κB signaling pathway, were administered for 1 h prior to stimulation with TNF-α. Inflammatory factors, such as IL-1β, IL-6 and IL-8, were investigated. As shown in Fig. 3A, TNF-α stimulation increased the mRNA expression of IL-1β, IL-6 and IL-8, and coincubation with SC-514 or DXM decreased the expression of IL-1β, IL-6 and IL-8. To assess the potential mechanism underlying the function of miR-146a in HCEC inflammation, we evaluated the expression of miR-146a after stimulation with TNF-α and coincubation with SC-514 or DXM. The RT‒qPCR results showed that the expression of miR-146a was upregulated by TNF-α stimulation, and this effect was reversed by coincubation with SC-514 or DXM (Fig. 3B). These data suggested that miR-146a played an important role in HCEC inflammation.

MiR-146a targets IRAK1and TRAF6 to regulate inflammation through the NF-κB signaling pathway in many tissues^17–19^. Intercellular adhesion molecule 1(ICAM1) and COX2 are two inflammatory genes and are downstream of the NF-κB signaling pathway. The mRNA expression of IRAK1, TRAF6, COX2 and ICAM1 was analyzed by RT‒qPCR (Fig. 3C). The results indicated that the mRNA expression levels of IRAK1, TRAF6, COX2 and ICAM1 were upregulated by stimulation with TNF-α and downregulated by coincubation with SC-514 or DXM.

### The target genes IRAK1 and TRAF6 are negatively regulated by miR-146a during HCEC inflammation

**Figure 4:**
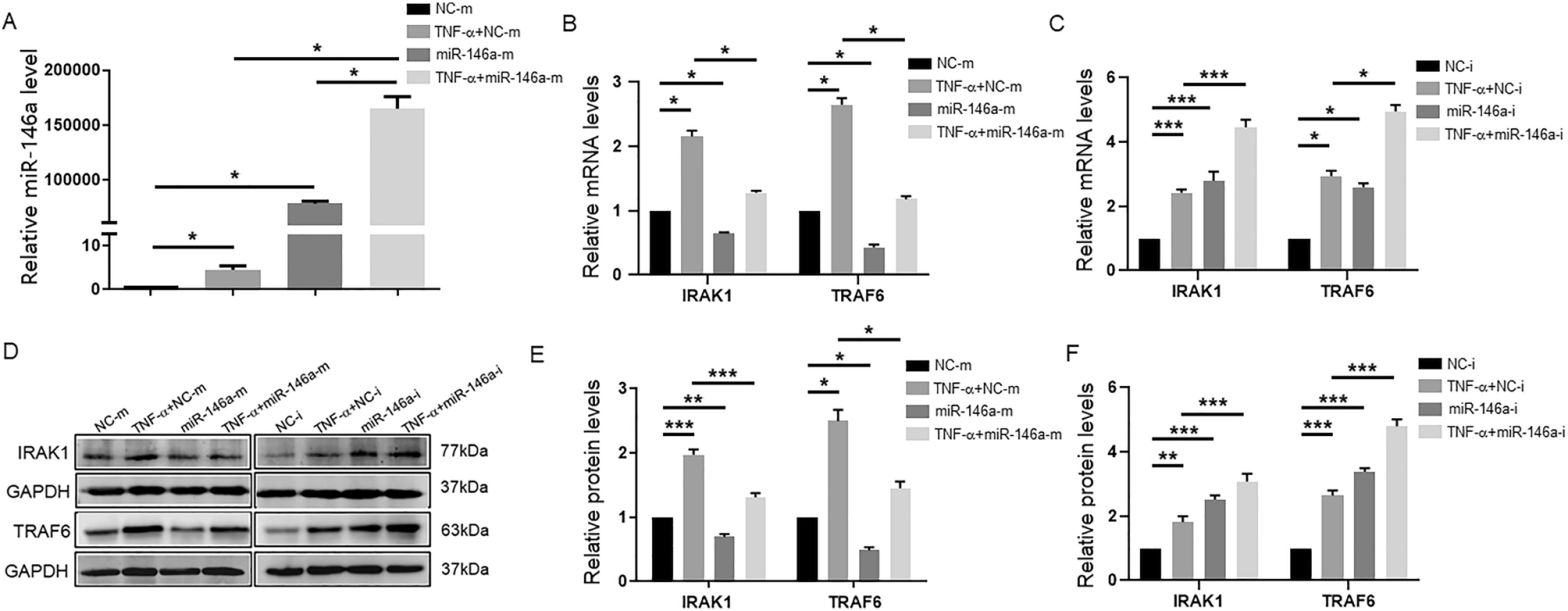
IRAK1 and TRAF6 are negatively regulated by miR-146a during HCEC inflammation. HCECs were transfected with miR-146a mimic (miR-146a-m), negative control (NC-m), miR-146a inhibitor (miR-146a-i) and inhibitor negative control (NC-i) absence or presence of TNF-α, respectively. (**A**) The expression of miR-146a was measured by RT‒qPCR in the NC-m group, the TNF-α + NC-m group, the miR-146a-m group and the TNF-α + miR-146a-m group. U6 was used as an endogenous control. (**B**) The mRNA expression levels of IRAK1 and TRAF6 were measured by RT-qPCR in the NC-m group, the TNF-α + NC-m group, the miR-146a-m group and the TNF-α + miR-146a-m group. GAPDH was used as an endogenous control. (**C**) The mRNA expression levels of IRAK1 and TRAF6 were measured by RT-qPCR in the NC-i group, the TNF-α + NC-i group, the miR-146a-i group and the TNF-α + miR-146a-i group. (**D**) The protein expression levels of IRAK1 and TRAF6 were detected by western blotting in HCECs transfected with miR-146a-m, NC-m, miR-146a-i and NC-i absence or presence of TNF-α, and the quantified data are shown in (E, F). The results are expressed as the mean ± SD. *P < 0.05, **P < 0.01, ***P < 0.001.

To understand the biological function of miR-146a, HCECs were transfected with a miR-146a mimic (agomir oligonucleotides) and inhibitor (antagomir oligonucleotides) in order to perform gain-of-function and loss-of-function experiments. The expression level of miR-146a in the mimic (miR-146a-m) group was elevated by 78,317-fold, as confirmed by RT‒qPCR analysis, compared with that in the negative control (NC-m) group. Consistent with this, the expression of miR-146a was increased by 4.49-fold in the TNF-α + NC-m group compared with the NC-m group, and the expression of miR-146a in the TNF-α + miR-146a-m group was also elevated by 36,748-fold compared with that in the TNF-α + NC-m group (Fig. 4A). These data indicated that the miR-146a mimic had been successfully transfected into HCECs.

IRAK1 and TRAF6 were confirmed to be target genes of miR-146a in previous studies^17–19^. Therefore, we next investigated the expression of IRAK1 and TRAF6 in HCECs transfected with the miR-146a mimic (miR-146a-m) and inhibitor (miR-146a-i). As shown in Fig. 4B–F, the expression of IRAK1 and TRAF6 was downregulated at both the mRNA and protein levels in the miR-146a-m group compared with the control (NC-m group). Moreover, after TNF-α stimulation, the expression of IRAK1 and TRAF6 was decreased in the miR-146a overexpression group. Consistent with these results, the expression of IRAK1 and TRAF6 in the miR-146a-i group was upregulated compared with that in the control group (NC-i group) at both mRNA and protein levels both in the presence and absence of TNF-α treatment. These results indicated that IRAK1 and TRAF6 can be negatively regulated by miR-146a during HCEC inflammation.

### MiR-146a negatively regulates the inflammatory response in HCECs through the NF-κB pathway

**Figure 5:**
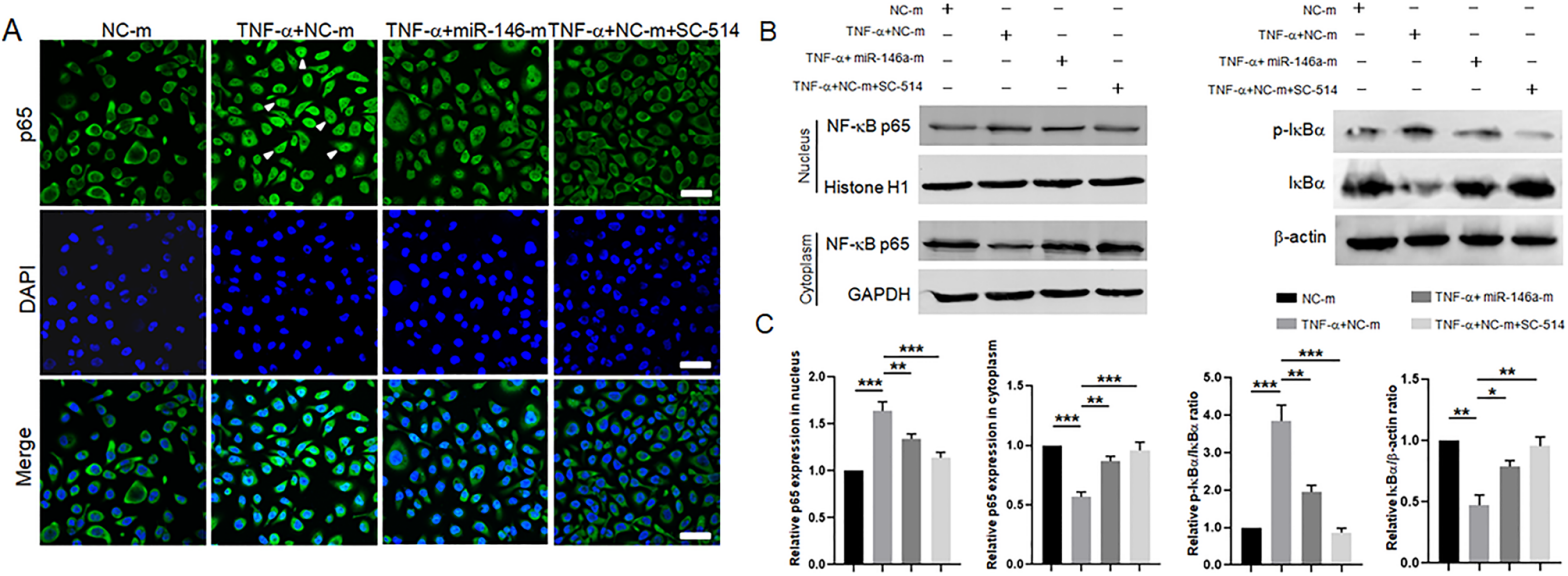
The NF-κB pathway is negatively regulated by miR-146a during HCEC inflammation. (**A**) The NF-κB p65 nuclear translocation was assessed by the immunofluorescence staining in the NC-m group, the TNF-α + NC-m group, the TNF-α + miR-146a-m group and the TNF-α + NC-m + SC-514 group. *Scale bars*: 50 μm. (**B**) The protein expression levels of NF-κB p65 and IκBα were measured by western blotting in the NC-m group, the TNF-α + NC-m group, the TNF-α + miR-146a-m group and the TNF-α + NC-m + SC-514 group and the quantified data are shown in (**C**). The results are expressed as the mean ± SD. *P < 0.05, **P < 0.01, ***P < 0.001.

As shown in the results described above, miR-146a targeted IRAK1 and TRAF6 during HCEC inflammation. IRAK1 and TRAF6 are two important adaptors and activators that act upstream of the NF-κB pathway. To further analyze the regulatory effect of miR-146a on the NF-κB pathway, we used the inhibitor SC-514 (20 μM) to block the NF-κB pathway. As shown by the immunofluorescence analysis, NF-κB p65 translocated from the cytoplasm to the nucleus to a greater extent in the TNF-α + NC-m group than in the NC-m group. The amount of NF-κB p65 that translocated to the nucleus was decreased in the TNF-α + miR-146a-m group compared with the TNF-α + NC-m group. NF-κB p65 translocation to the nucleus was suppressed in the TNF-α + NC-m + SC-514 group compared with the TNF-α + NC-m group (Fig. 5A). Moreover, the western blotting analysis results were consistent with the immunofluorescence analysis results. TNF-α stimulation caused an increase in the nuclear accumulation of NF-κB p65 and a decreased in the level of cytosolic NF-κB p65, whereas overexpression of miR-146a or SC-514 treatment blocked these effects (Fig. 5B,C). In addition, NF-κB activity is prevented by nuclear factor kappa B (IκB)α proteins. Upon stimulation with an inducer, such as TNF-α or LPS, IκBα is phosphorylated, degradated and dissociated from NF-κB, resulting in NF-κB translocation to the nucleus. We therefore measured the IκBα protein levels in the cytoplasm by using western blotting. TNF-α treatment caused IκBα phosphorylation and degradation, while overexpression of miR-146a in HCECs attenuated TNF-α induced IκBα phosphorylation and degradation. Collectively, these results suggest that miR-146a may inhibit NF-κB activity by targeting TRAF6 and IRAK1.

### MiR-146a negatively regulates inflammatory factors downstream of the NF-κB pathway during HCEC inflammation

**Figure 6:**
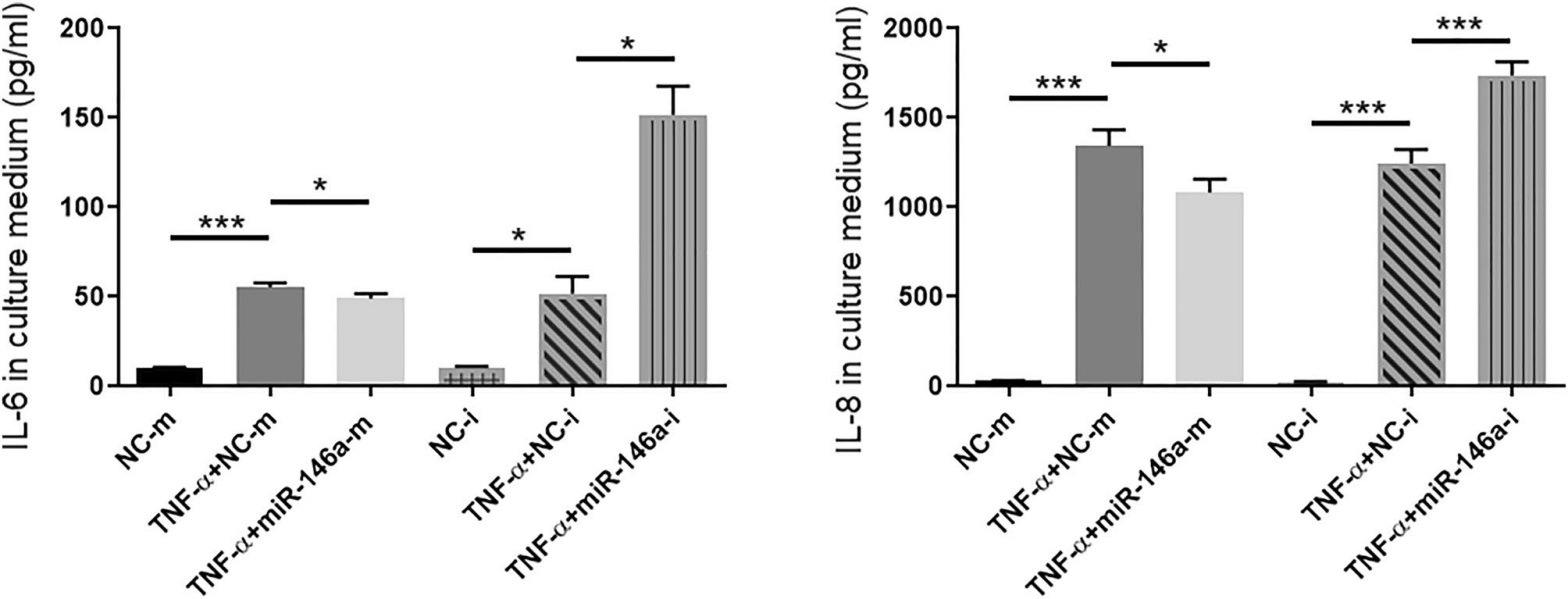
Inflammatory factors IL-6 and IL-8 are negatively regulated by miR-146a during HCEC inflammation. MiR-146a was overexpressed or inhibited in HCECs by transfection with miR-146a mimic or miR-146a inhibitor in the presence of TNF-α. The secretion of the IL-6 and IL-8 proteins in cell culture supernatant was measured by ELISA. The results are expressed as the mean ± SD. *P < 0.05, **P < 0.01, ***P < 0.001.

IL-6 and IL-8 are two inflammatory factors that are regulated by the NF-κB signaling pathway. The levels of IL-6 and IL-8 proteins that were secreted into cell culture supernatants were measured by ELISA. The results showed that the secretion of the IL-6 and IL-8 proteins was significantly increased upon stimulation with TNF-α The secretion of the IL-6 and IL-8 proteins in TNF-α + miR-146a-m group was decreased compared with that in the TNF-α + NC-m group, whereas the secretion of the IL-6 and IL-8 proteins in the TNF-α + miR-146a-i group was increased compared with that in the TNF-α-NC-i group (Fig. 6). These data suggested that overexpression of miR-146a decreased the secretion of the IL-6 and IL-8 proteins, while inhibition of miR-146a increased the secretion of the IL-6 and IL-8 proteins. IL-6 and IL-8 secretion was negatively regulated by miR-146a during HCEC inflammation.

**Figure 7:**
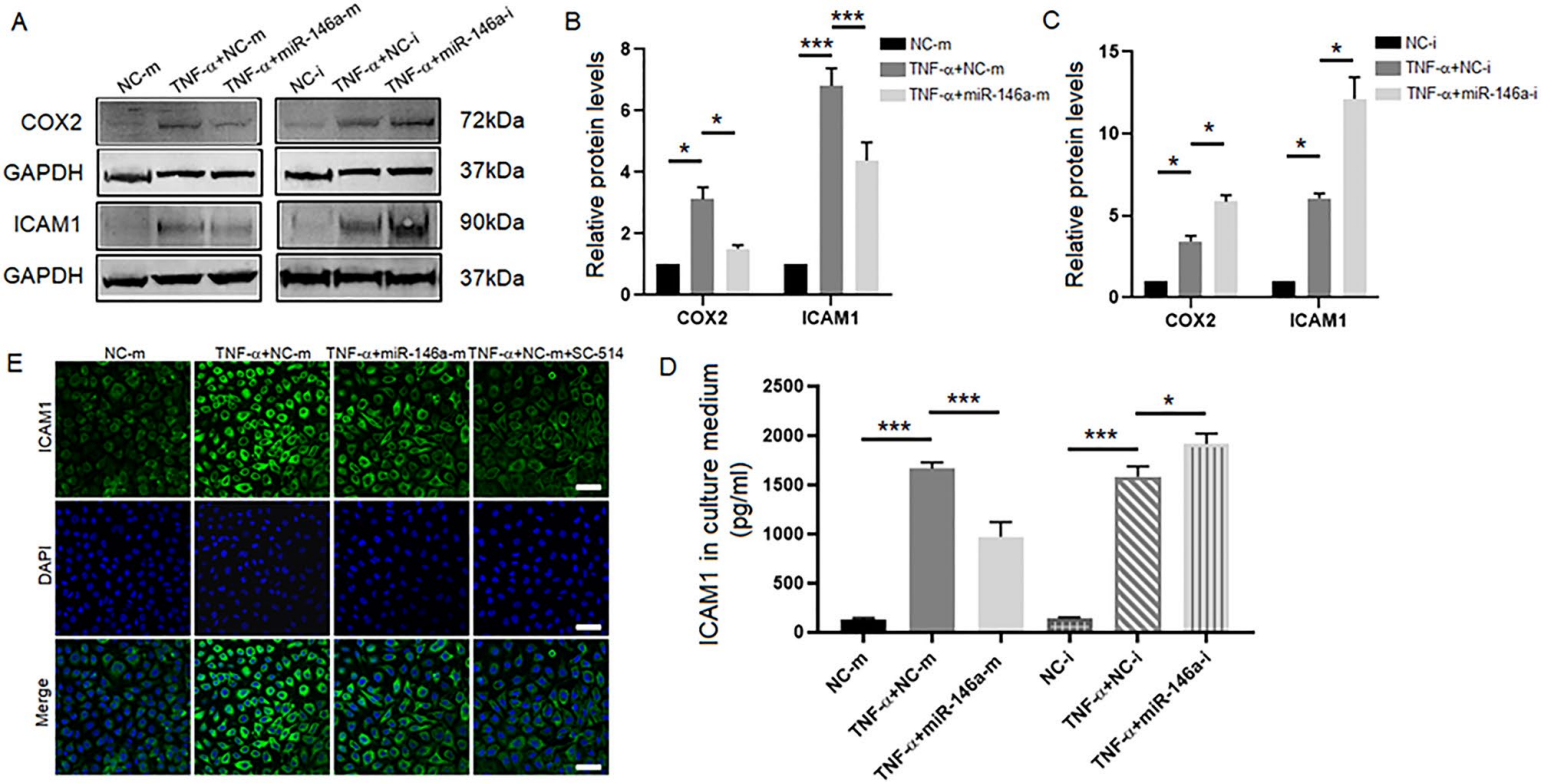
COX2 and ICAM1 are negatively regulated by miR-146a during HCEC inflammation. (**A**) The protein levels of COX2 and ICAM1 were measured by western blotting in the NC-m group, the TNF-α + NC-m group, the TNF-α + miR-146a-m group, the NC-i group, the TNF-α + NC-i group and the TNF-α + miR-146a-i group. The quantified data are shown in (**B**) and (**C**). (**D**) The secretion of sICAM1 protein in cell culture supernatant was measured by ELISA in the NC-m group, the TNF-α + NC-m group, the TNF-α + miR-146a-m group, the NC-i group, the TNF-α + NC-i group and the TNF-α + miR-146a-i group. The results are expressed as the mean ± SD. *P < 0.05, **P < 0.01, ***P < 0.001. (**E**) The protein expression of ICAM1 was showed by the immunofluorescence staining in the NC-m group, the TNF-α + NC-m group, the TNF-α + miR-146a-m group and the TNF-α + NC-m + SC-514 group. *Scale bars*: 50 μm.

ICAM1 is a cell surface glycoprotein and an adhesion receptor. COX2 is an enzyme of secondary inflammatory mediators. ICAM1 and COX2 are two downstream genes of the NF-κB signaling pathway^28^. Western blotting results showed that the protein levels of COX2 and ICAM1 were upregulated upon stimulation with TNF-α. The expression of COX2 and ICAM1 in the TNF-α + miR-146a-m group was downregulated compared with that in the TNF-α + NC-m group (Fig. 7A,B), and the expression levels of COX2 and ICAM1 in the TNF-α + miR-146a-i group were upregulated compared with those in the TNF-α + NC-i group (Fig. 7A,C). In contrast, inhibition of miR-146a increased the protein expression of COX2 and ICAM1 in HCECs. These results indicated that overexpression of miR-146a downregulated the protein expression levels of COX2 and ICAM1.

ICAM1 is present in its soluble form (sICAM1) in numerous inflammatory disorders, and sICAM1 is produced as a spliced isoform or as a result of proteolytic cleavage^29^. The levels of the sICAM1 protein that were secreted into cell culture supernatants were measured by ELISA. The ELISA results showed that the secretion of the sICAM1 protein was increased upon stimulation with TNF-α. The secretion of the sICAM1 protein in the TNF-α + miR-146a-m group was reduced compared with that in the TNF-α + NC-m group, while the secretion of the sICAM1 protein in the TNF-α + miR-146a-i group was increased compared with that in the TNF-α + NC-i group (Fig. 7D). The ELISA results were consistent with the western blotting results described above. As shown by immunofluorescence analysis (Fig. 7E), the expression of ICAM1was upregulated under TNF-α stimulation, miR-146a-m cotreatment reduced the expression of ICAM1, and SC-514 cotreatment reduced the expression of ICAM1. These results showed that miR-146a negatively regulated the inflammatory factors IL-6, IL-8, COX2 and ICAM1, which are downstream genes of the NF-κB pathway, in HCECs.

## Discussion

Dry eye disease (DED) is a heterogeneous disorder of the ocular surface that is characterized by a loss of tear film homeostasis and accompanied by symptoms including tear film instability, hyperosmolarity, and inflammation^1,2^. Integrated tear proteomic and metabolomic analyses have revealed that inflammatory proteins and metabolites play important roles in the development of DED^30^. Inflammation is the common denominator in DED, and it is both a cause and effect of the disease^5,31^.

In the current study, we established a dry eye mouse model via the administration of BAC. BAC accumulation induces a reduction in mucins and an alteration of the lipid layer, leading to impairments of the tear film with tear instability and excessive evaporation, which are hallmarks of dry eye disease^1,25^. Studies have revealed that BAC induces high expression of proinflammatory mediators in the cornea and conjunctiva^32,33^. In this study, the expression of the proinflammatory factors TNF-α and IL-1β was increased and the classic NF-κB pathway was activated in the cornea. In the future, various dry eye models should be established to further verify the role of miR-146a in DED.

Several miRNAs have been shown to be involved in the regulation of corneal inflammation^34,35^. Overexpression of miR-328 may contribute to DED, and anti-miR-328 treatment protects corneal cells and promotes re-epithelialization in the treatment of DED^34^. MiR-146a plays a key role in the regulation of innate immunity and inflammation^11–14^. There was no significant difference in the expression of miR-146a in the blood of patients with dry eye disease and healthy people^36^, but the expression of miR-146a in the corneas of patients with dry eye disease and healthy people is not clear. In the present study, we explored the role of miR-146a in corneal inflammation using a dry eye mouse model and HCEC inflammation model.

The upregulated expression of miR-146a in the corneas of dry eye model mice suggested that miR-146a may play a role in corneal inflammation. We treated HCECs with TNF-α, a proinflammatory cytokine, to establish an inflammatory model in HCECs. To gain an in-depth understanding of the biological functions of miR-146a, HCECs were transfected with a miR-146a mimic and inhibitor in order to perform gain-of-function and loss-of-function experiments. IRAK1 and TRAF6 have been confirmed to be the unequivocal target genes of miR-146a in several studies^17–19^. The expression of IRAK1 and TRAF6 was upregulated in the corneas of dry eye model mice and during HCEC inflammation. Overexpression of miR-146a decreased the protein levels of IRAK1 and TRAF6 in HCECs, while inhibition of miR-146a increased the expression of IRAK1 and TRAF6. These results demonstrate that miR-146a plays an important role in the regulation of corneal epithelial cells inflammation via targets IRAK1 and TRAF6 during dry eye disease.

The NF-κB pathway is an important inflammatory pathway, and dysregulated NF-κB activity causes inflammation-related diseases as well as cancers; NF-κB has been proposed as a potential target for disease therapy^37,38^. Western blotting results showed that the NF-κB pathway was activated in the dry eye mouse model. IRAK1 and TRAF6 are two well-known activators of the NF-κB pathway^39^. To further analyze the regulatory effect of miR-146a on the NF-κB signaling pathway during HCEC inflammation, we used the NF-κB pathway inhibitor SC-514 to block the NF-κB pathway^40,41^. The results showed that TNF-α induced the translocation of NF-κB p65 from the cytoplasm to the nucleus, while cotreatment with SC-514 inhibited the translocation of NF-κB p65 from the cytoplasm to the nucleus and inhibited the activation of the NF-κB pathway. Similarly, cotreatment with the miR-146a mimic reduced NF-κB p65 translocation to the nucleus. The results indicated that miR-146a targets IRAK1 and TRAF6 to regulate HCEC inflammation through the NF-κB pathway. A recent study reported that miR-146a targets IRAK1 and TRAF6 during lipopolysaccharide (LPS)-induced acute inflammation in corneal limbal epithelium cells (LECs), and overexpression of miR-146a decreased the expression of TRAF6 and IRAK1 and the downstream target NF-κB after challenge with LPS or wounding^42^.

Studies have shown that miR-146a negatively regulates the NF-κB signaling pathway during inflammation in different tissues^12,14^. MiR-146a is upregulated in diabetic corneas, especially in corneal limbus, and this could be a defense mechanism to prevent acute or diabetic chronic inflammation by acting through its target proteins TRAF6 and IRAK1^42^. Our data are consistent with theirs. We show that miR-146a is upregulated in the corneas of dry eye mouse model (Fig. 1). Furthermore, miR-146a is induced by TNF-α in a NF-κB activation dependent manner in HCECs (Fig. 3). Besides, both gain-of-function and loss-of-function experiments demonstrate that miR-146a inhibits the activation of NF-κB through the targets of TRAF6 and IRAK1, and results in the decrease of inflammatory factors production in HCECs (Figs. 4, 5, 6, 7). These suggest that there might be a negative feedback regulation between miR-146a upregulation and NF-κB activation in the inflammatory response during dry eye disease, by which exerts an endogenous protective mechanism for controlling the excessive inflammatory response. Studies in other area also have shown that miR-146a acts in a negative feedback loop to regulate NF-κB activation. An important role of miR-146a was observed in models of diabetic retinopathy. Thrombin treatment results in NF-κB-dependent upregulation of miR-146 in human retinal microvascular endothelial cells (HRECs). MiR-146 can inhibit thrombin-induced NF-κB activation and increased leukocyte adhesion to HRECs by suppressing its target protein caspase-recruitment domain (CARD)-containing protein 10 (CARD10), suggesting a negative feedback regulation on thrombin-induced NF-κB activation^43^. The expression of miR-146a is upregulated during the replicative senescence of human trabecular meshwork cells, and the upregulation of miR-146a may decrease the production of inflammatory mediators such as IL-6 and IL-8 and intracellular reactive oxygen species in order to inhibit the excessive production of inflammatory mediator in senescent cells and limit their deleterious effects on the surrounding tissues^44^.

COX2 is an inducible enzyme whose expression is induced by inflammatory mediators and exerts proinflammatory effect. ICAM1 is a transmembrane glycoprotein that belongs to the immunoglobulin supergene family. NF-κB acts in concert with transcription factors or transcription coactivators to mediate the induction of ICAM1 and COX2 expression^28^. Our study demonstrated that miR-146a mimics can reduce the expression of COX2 and ICAM1, while miR-146a inhibitors can increase the expression of COX2 and ICAM1. MiR-146a negatively regulated the expression of IL-6, IL-8, COX2 and ICAM1, which are downstream genes of the NF-κB pathway, during HCEC inflammation. The function of ICAM1 is typically associated with promoting inflammation; however, emerging evidence increasingly implicates ICAM1 in the resolution of inflammation and tissue healing^45,46^. Soluble ICAM1 secreted by human umbilical cord blood-derived mesenchymal stem cells reduces amyloid-β plaques^47^. ICAM1 mediates surface contact between neutrophils and keratocytes following corneal epithelial abrasion in mice^48^. The regulatory effect of ICAM1 on corneal epithelial inflammation needs to be further studied.

The etiology and pathogenesis of DED is complex and multifactorial. Tear film instability, tear film hyperosmolarity, ocular surface damage and ocular surface inflammation are the key events in the pathogenesis of the disease. MiR-146a has shown that it is extensively involved in the innate immune system as a negative regulator of inflammation. However, the effect of miR-146a wound healing remains uncertain. Poe et al. has reported that several identified miR-146a-targeted pathways are important for corneal epithelial homeostasis, such as EGFR, Notch, anchoring junctions, adherens junctions, TGF-β and inflammation-related signaling^49^. Our present study shows a negative feedback regulation of miR-146a on NF-κB activation and its downstream secreted inflammatory cytokines via IRAK1 and TRAF6 in HCECs, suggesting a potential therapeutic effect on controlling inflammation during DED. Therefore, it is necessary to verify the effect of miR-146a mimic on animal dry eye model in the future.

In summary, our study shows that miR-146a plays an important role in the regulation of HCEC inflammation and corneal inflammation. MiR-146a may target IRAK1 and TRAF6 and negatively regulate HCEC inflammation through the NF-κB pathway. Therefore, the miR-146a/IRAK1/TRAF6/NF-κB pathway can be used as a potential therapeutic target for DED treatment.

## Materials and methods

### Dry eye mouse model

Healthy 5-week-old male BALB/c mice were purchased from the Department of Hematology, Peking Union Medical College. Forty mice with normal ocular surface were screened by slit lamp. All experimental procedures involving animals were performed in accordance with the Association for Research in Vision and Ophthalmology (ARVO) Statement for the Use of Animals in Ophthalmic and Vision Research. This study was approved by the Institutional Animal Care and Use Committee of Tianjin Medical University and carried out in compliance with the ARRIVE guidelines. The mice were housed in a 12 h light/12 h dark cycle at 25 °C ± 1 °C with 60% ± 10% humidity. The mouse dry eye model was induced by BAC^24,25^. The right eyes of BALB/c mice were treated with twice-daily (9 A.M., 9 P.M.) topical administration of 5 μl of 0.2% BAC (Sigma-Aldrich, USA) for 14 days. The contralateral eyes were treated with PBS as controls. The eye balls were collected from the mice which were killed by cervical dislocation.

### Tear film break up time (BUT) and fluorescein staining

Fluorescein sodium (1%) was dropped into the conjunctival sac, and the eyelids were manually closed 3 times. At the first appearance of a dry sign, BUT was recorded in seconds. The testing time was recroded under a slit lamp microscope, and the BUT test was repeated 3 times for each eye. Ninety seconds later, corneal epithelial damage was graded with a cobalt blue filter under a slit-lamp microscope. The cornea was divided into 4 quadrants, which were scored, respectively. The 4 scores were added to arrive at a final grade (total, 16 points).

### Cell culture

SV40 immortalized human corneal epithelial cell line was provided by Dr. Zhang Yan (Tianjin Medical University Eye Hospital). The cell line was authenticated by Short Tandem Repeat (STR) Genotyping method. The cells were cultured in DMEM/F12 supplemented with 6% fetal bovine serum (Gibco), 1% penicillin (100 U/ml, Gibco), 1% streptomycin (100 μg/ml, Gibco), 7 μg/ml insulin (Sigma-Aldrich) and 7 ng/ml epithelium growth factor (Gibco)^50,51^. Cells were incubated in a humidified 5% CO_2_ incubator at 37 °C. For TNF-α treatment, the cells were grown in six-well plates and treated with 10 ng/ml recombinant human TNF-α.

### Transfection of microRNA

The miR-146a-5p agomir, negative control (NC-m), miR-146a-5p antagomir and inhibitor negative control (NC-i) were obtained from GenePharma (Shanghai, China). The agomir of miR-146a-5p used for overexpression was double stranded with the following sequence: 5′-UGAGAACUGAAUUCCAUGGGUU-3′ (forward) and 5′-CCCAUGGAAUUCAGUUCUCAUU-3′ (reverse). The miR-146a-5p negative control: 5′-UUCUCCGAACGUGUCACGUTT-3′ (forward) and 5′-ACGUGACACGUUCGGAGAATT-3′ (reverse). The miR-146a-5p antagomir was single stranded with the following sequence: 5′-AACCCAUGGAAUUCAGUUCUCA-3′ and inhibitor negative control with the following sequence: 5′-CAGUACUUUUGUGUAGUACAA-3′. HCECs were transfected with miR-146a-5p agomir/antagomir or NC-m /NC-i using Lipofectamine 3000 Transfection Reagent (Invitrogen, Carlsbad, CA) accroding to the manufacturer’s instructions. HCECs were cultured in a 6-well plate (1 × 10^5^ cells/well) approximately 60–80% confluence at the time of transfection. Twenty-four hours after transfection, TNF-α (10 ng/ml) was added or not to cell medium.

### RNA extraction and real-time quantitative PCR

Corneal tissues (n = 4 samples per group) and cells total RNA was extracted using a total RNA purification kit (TianGen, Beijing, China). The amount of total RNA was quantified by spectrophotometry and cDNA was synthesized with M-MLV reverse transcriptase (Promega, Madison, USA). Real-time quantitative PCR (RT‒qPCR) was performed with SYBR Primix Ex Taq (Takara, Japan) and the StepOnePlus Real-Time PCR system (Applied Biosystems, CA, USA) according to the manufacture’s protocol. The relative quantity of mRNA or miR-146a with regard to the expression of GAPDH or U6 was estimated using the 2^−△△Ct^ method.

### Enzyme-linked immunosorbent (ELISA) assay

The concentrations of the inflammatory cytokines IL-6, IL-8 and sICAM1 in culture supernatants were determined using ELISA Kit (Joyee Biotechnics, Anhui, China). HCECs were seeded in 6-well plate and transfected with miR-146a-5p oligonucleotides for 24 h. TNF-α was added or not to cell medium for 24 h. Then the culture supernatants were collected for measurement of IL-6, IL-8 and sICAM1. All assays were performed according to the manufacture’s protocol.

### Western blot analysis

Corneal tissues (n = 4 samples per group) were ground and homogenized in RIPA buffer and cell lysates were prepared with RIPA buffer (Beyotime Institute of Biotechnology, Shanghai, China) containing a protease inhibitor cocktail (sigma-aldrich, USA) and proteins were harvested after centrifugation. NF-κB protein was prepared with Nuclear and Cytoplasmic Extraction Reagents kit (Thermo Fisher Scientific, Rockford, USA). The protein concentration was determined using BCA protein assay kit (Thermo, Rockford, USA). Proteins (20–30 μg per lane) were separated by SDS-PAGE and transferred to a polyvinylidene difluoride (PVDF) membrane (Millipore, Bedford, MA, USA). The membranes were blocked in 5% nonfat dry milk in PBS for 1 h and then incubated with primary antibodies against IRAK1 (1:1000, ab238, Abcam), TRAF6 (1:1000, ab33915, Abcam,), COX2 (1:1000, ab179800, Abcam), ICAM1(1:1000, ab109361, Abcam), NF-κB p65 (1:1000, ab32536, Abcam), IKBα (1:1000, ab76429, Abcam), p-IKBα (1:1000, ab133462, Abcam), β-actin (1:1000, ab8227, Abcam) and GAPDH (1:1000, ab181602, Abcam) at 4 °C overnight. Subsequently, the membrane were washed by TBST and incubated with secondary antibodies (dilution 1:10,000, Abcam, USA) at room temperature for 2 h. Following washing with TBST for three times, the protein bands were detected by the Odyssey Sa Two-colour infrared laser imaging system (licor, USA). The image was analyzed by using ImageJ software.

### Immunofluorescence staining

HCECs were fixed with 4% formaldehyde for 20 min at room temperature (RT), permeated with 0.5% Triton X-100 for 10 min and blocked with 5% bovine serum albumin (BSA) for 1 h. Afterwards, the cell slides were incubated with primary antibody against NF-κB p65 (1:100, ab32536, Abcam), ICAM1 (1:100, ab109361, Abcam) overnight at 4 °C. Next day, secondary antibody Alexa Fluor 488-conjugated goat anti-rabbit (Thermo Fisher Scientific, USA) incubated for 1 h at RT. After washing with PBS, the cell slides were incubated with DAPI for nuclear staining and mounted with anti-fade mounting medium. The slides were observed using a laser scanning confocal microscopy (Leica, TCS SP8 SR, Germany).

### Histological and immunohistochemical staining

The whole eyeballs were immersed in 4% paraformaldehyde for 24 h at room temperature. Subsequently, the eyeballs were treated with gradient alcohol and xylene and embedded in paraffin for sectioning at a thickness of 4 μm. Tissue sections stained with hematoxylin/eosin were used to observe the corneal structure. Immunohistochemical staining was performed according to our previously published protocol^52^. Briefly, antigen retrieval was performed with sodium citrate repair solution by microwave heating, non-specific antigens were eliminated by 3% H_2_O_2_, and the sections were blocked with goat serum for 1 h and incubated with primary antibodies against IL-1β (1:100, ab254360, Abcam), TNF-α (1:1000, ab183218, Abcam), TRAF6 (1:300, ab33915, Abcam), IRAK1 (1:1000, ab238, Abcam), and COX2 (1:400, ab179800, Abcam) overnight at 4 °C. Next day, the sections were incubated with the Envision horseradish peroxidase system (Gene Tech, Shanghai, China) for 2 h at room temperature. The sections were finally incubated with 3,3′-diaminobenzidine (DAB) (Gene Tech, Shanghai, China) for 5 min. The staining was observed and captured with an Eclipse Ni microscope (Nikon, Tokyo, Japan).

### Statistical analysis

All quantitative data were shown as mean ± SD, and statistical analyses were performed with SPSS version 20 (IBM Corp., Armonk, NY, USA). The results presented in the figures were expressed at least three independent experiments. The data were tested for normality with the Shapiro Wilk test or the Kolmogorov–Smirnov test. A Student t-test was used to assess the differences between the two means. The assessment of multiple means was performed by one-way ANOVA followed by the Bonferroni’s post hoc test. In case of a low sample size (low n number) that was not sufficient for normality testing, nonparametric tests (the Mann–Whitney U test or Kruskal–Wallis test) were used. Statistical significance was accepted at *P* < 0.05.

## Data Availability

All data generated or analyzed during this study are included in this published article.
